# Magnitude and Predictors of Leukopenia and Thrombocytopenia in Adults With HIV/AIDS Attending Mizan Tepi University Teaching Hospital, Southwest Ethiopia

**DOI:** 10.1155/bmri/5907903

**Published:** 2026-04-10

**Authors:** Samuel Sahile Kebede, Dereje Zeleke, Tariku Belay, Eyob Atilabachew, Getachew Mesfin Bambo

**Affiliations:** ^1^ Department of Medical Laboratory Sciences, College of Medicine and Health Sciences, Mizan Tepi University, Mizan Aman, Ethiopia, mtu.edu.et; ^2^ Department of Pediatrics and Child Health, College of Medicine and Health Sciences, Mizan Tepi University, Mizan Aman, Ethiopia, mtu.edu.et; ^3^ Department of Public Health, Mizan Aman College of Health Sciences, Mizan Aman, Ethiopia; ^4^ Department of Internal Medicine, College of Medicine and Health Sciences, Mizan Tepi University, Mizan Aman, Ethiopia, mtu.edu.et

**Keywords:** CD4, leukocytopenia, lymphocytopenia, thrombocytopenia, viral load

## Abstract

**Background:**

Leukopenia and thrombocytopenia are common hematological problems among individuals living with human immunodeficiency virus (HIV/AIDS), with a reported prevalence of 6%–43.4%. Despite their clinical and public health significance, there is a limited number of studies done on their magnitude and associated predictors.

**Objective:**

This study was aimed at assessing the magnitude and predictors of leukopenia and thrombocytopenia among adults with HIV/AIDS at Mizan Tepi University Teaching Hospital (MTUTH), Southwest Ethiopia.

**Methods:**

An institution‐based analytical cross‐sectional study was carried out among adults living with HIV/AIDS attending MTUTH, Southwest Ethiopia, from March to April 2023. A semistructured questionnaire was used to obtain sociodemographic and clinical data. Five milliliters of venous blood sample was collected from each participant in EDTA tubes and analyzed using the UniCel DxH 800 hematology analyzer. CD4+ T‐cell counts were measured using the BD FACS flow cytometer, while HIV‐1 viral load was assessed using the GeneXpert HIV‐1 PCR assay. Data was entered into EpiData 4.6 and analyzed by STATA 14. Descriptive statistics were computed, and multivariable logistic regression was used to assess determinants at *p* < 0.05.

**Result:**

The study enrolled 358 participants. The magnitude of leukopenia, thrombocytopenia, and anemia was 14.8% (95% CI: 13.75–15.85), 10.34% (95% CI: 9.34–11.34), and 36.9% (95% CI: 35.90–37.90), respectively. Leukopenia was significantly associated with age > 65 years (AOR = 2.80; 95% CI: 1.41, 3.36; *p* = 0.001), pregnancy (AOR = 3.92; 95% CI: 1.76, 8.71; *p* = 0.04), MTB (AOR = 3.67; 95% CI: 1.89, 7.12; *p* = 0.001), viral load (AOR = 5.12; 95% CI: 3.01, 8.19; *p* = 0.046), and CD4+ (AOR = 4.32; 95% CI: 2.02, 7.45). Thrombocytopenia was significantly associated with age > 65 years (AOR = 2.2; 95% CI: 1.72–2.72; *p* = 0.009), hypertension (AOR = 4.1; 95% CI: 3.63, 4.63; *p* = 0.03), undernutrition (AOR = 5.42; 95% CI: 4.82, 5.92; *p* = 0.007), and alcohol consumption (AOR = 4.21; 95% CI: 4.01, 4.31; *p* = 0.006).

**Conclusion and Recommendation:**

Among HIV patients, leukocytopenia and thrombocytopenia are common public health problems that have been associated with age > 65 years, pregnancy, MTB infection, undernutrition, hypertension, alcohol consumption, high viral load, and low CD4+ count. To improve clinical outcomes and reduce HIV‐related problems, routine blood count monitoring and targeted management of high‐risk patients are recommended.

## 1. Introduction

Hematological abnormalities are the common abnormalities that individuals affected by human immunodeficiency virus and acquired immunodeficiency syndrome (PLWHIV/AIDS) have, which are correlated with hematological and immunological abnormalities [1]. As human immunodeficiency virus (HIV) causes acquired immunodeficiency syndrome, immunological and hematological parameters become decreased due to bone marrow depression. The bone marrow suppression in patients with HIV leads to pancytopenia, characterized by a significant decrease in the cluster of differentiation (CD4) count, as well as a decrease in platelets and leukocytes, including neutrophils, lymphocytes, monocytes, eosinophils, and basophils, which become both quantitatively and qualitatively decreased [2].

In HIV patients, the opportunistic infection and HIV itself result in hematological derangements by destruction of blood cells and inhibition of blood cell formation due to bone marrow suppression [3]. The T‐cells primarily recognize antigens through the T‐cell receptor and play key roles in inflammation, stimulating macrophage phagocytosis, promoting B‐cell proliferation during antibody production, and identifying infected cells for destruction. As the disease progresses, due to the destruction of cells, further complications such as thrombocytopenia, thrombotic thrombocytopenic purpura (TTP), leukopenia, lymphopenia, thrombosis, malignancy, and infections can occur. As a result, individuals with HIV are at risk of illness and death from opportunistic infections, cancers, and related complications [4, 5].

Leukopenia and thrombocytopenia are major hematological problems next to anemia, which can happen as a consequence of HIV infection, as well as other opportunistic infections and different antivirals (especially AZT‐based regimens), pregnancy, increased age, hypertension, malnutrition (under‐ and overweight), high‐level alcohol consumption, and antibiotics [6].

Leukopenia can occur mainly due to decreased myeloid cells, especially neutrophils, and lymphoid cells (lymphocytes). Neutrophils are myeloid precursor cells that respond to both innate immune responses and pathogens. Since neutrophils are myeloid precursor cells that react to both innate immune responses and pathogens, they fight microorganisms, especially bacteria and fungi, by phagocytosing them and killing them by nicotinamide adenine dinucleotide phosphate (NADPH) oxidase or antibacterial proteins. They increase during acute infection and decrease in chronic HIV or bacterial coinfections. Its decrease significantly impacts the leukocyte count and causes leukopenia because it consumes a substantial amount of white blood cells [7, 8].

Among leukocytes, lymphocytes are major components, which are produced from lymphoid precursor cells and participate in adaptive immune responses. They contribute significantly to the immune response by recognizing and combating microorganism‐related infections. Decreased lymphocytes, especially CD4+ (T‐helper lymphocytes) cell counts, are also common in chronic viral infection (increased viral load). Therefore, decreased lymphocytes with low NLR can serve as a red flag alerting to a dysregulated immune system in HIV patients [8].

Thrombocytopenia is the first hematological sign that can be commonly seen in HIV infection, which leads to severe anemia due to bleeding in the increased HIV stage and deteriorated CD4 count. Severe thrombocytopenia restricts the use of medications for managing HIV infection and AIDS‐related cancers. The relationship between CD4 count and thrombocytopenia with low lymphocyte, NLR, and Hgb might help to use these parameters as an option in areas where it is difficult to get CD4 count and viral load to predict the immune status of an individual and the riskiness of a patient to malignancy and other complications [9].

Although leukopenia and thrombocytopenia are not considered serious problems in PLWHIV, they result in severe complications among HIV patients, such as severe bleeding, petechiae, bruising, infection, and death. It is important to research these hematological abnormalities [6]. Therefore, this study was aimed at assessing the magnitude of leukopenia and thrombocytopenia and their predictors among adults living with HIV.

## 2. Materials and Methods

### 2.1. Study Design and Duration

An institution‐based analytical cross‐sectional study was carried out to assess leukopenia and thrombocytopenia and their predictors among HIV‐infected adult patients attending MTUTH, Southwest Ethiopia, from March to April 2025.

### 2.2. Population

Source population: All HIV‐infected adults who were attending ART clinics at MTUTH, Southwest Ethiopia, were considered as the study population.

Study population: All adults living with HIV who attended the ART clinic at MTUTH during the data collection period were considered as the study population.

### 2.3. Inclusion Criteria and Exclusion Criteria

#### 2.3.1. Inclusion Criteria

All individuals aged 15 years and above with confirmed HIV infection who were up on follow‐up at MTUTH and with clinical records available within the 6 months prior to data collection were included in the study.

#### 2.3.2. Exclusion Criteria

Critically ill patients who were unable to provide responses or blood samples were excluded from the study.

### 2.4. Sample Size Calculation and Sampling Technique

The required sample size for the study was determined using the single population proportion formula, assuming a 50% proportion, a 95% confidence interval, and a 5% margin of error, as shown in the following statistical formula:

n=n=z2a/2 p1−Pd2,n=1.9620.510.5−0.052=384

where *n* is the sample size, *Z* is a level of confidence in the *Z*‐statistics, *P* is the expected proportion or prevalence, and *d* is the margin of error between the population and sample, where the population at follow‐up is less than 10,000 by using the population proportion formula:

n=no1+no/N,n=38413845190+/=358

### 2.5. Sampling Technique

Participants in the study were recruited by using a systematic random sampling technique. Twenty‐five HIV patients attended daily ART follow‐up appointments and provided a blood sample for both viral load and CD4+ T‐cell count at the same time. One thousand one hundred PLWHIV would visit the hospital for follow‐up on their CD4+ T‐cell count and viral load over the 2‐month data collection period. By dividing the total number of HIV/AIDS patients during our study period by the sample size (1100/358 = 3), the sampling interval (*K*) value was determined. After that, a lottery was used to select the first among three participants, who were thereafter chosen at intervals of three. Every three patients who visit the MTUTH ART clinic are selected as study subjects.

### 2.6. Methods and Tools for Data Collection

#### 2.6.1. Collection of Sociodemographic and Clinical Data

Data were collected using a semistructured questionnaire administered by trained nurses. The questionnaire had three parts, including sociodemographic and clinical data. Each tool was designed to ensure consistency, accuracy, and relevance of the collected information across all study participants and settings (Supporting Information 1: Annex S1 and Supporting Information 2: Annex S2).

#### 2.6.2. Blood Sample Collection and Hematological Testing

##### 2.6.2.1. Blood Sample Collection.

Approximately 5 mL of venous blood was collected from each participant using a sterile syringe and needle by a qualified medical laboratory technologist and transferred into an EDTA tube labeled with the participant′s code number. Samples were transported to the hematology laboratory within 1–2 h for analysis. Hematological parameters were measured first, followed by CD4+ T‐cell counts and HIV‐1 viral load testing from the remaining sample after plasma preparation (Supporting Information 3: Annex S3).

#### 2.6.3. Analysis of Hematological Parameters

Hematological analyses were performed on venous blood samples collected in EDTA anticoagulated tubes. Hgb concentration, erythrocyte count, platelet count, and leukocyte five‐part differential were measured using an automated UniCel DxH 800 hematology analyzer (Beckman Coulter, Danaher Corporation, United States). The analyzer determines erythrocyte and platelet counts using the electrical impedance (Coulter principle), in which cells are counted and sized based on changes in electrical resistance as they pass through a calibrated aperture. The analyzer distinguishes platelets from erythrocytes by evaluating differences in particle size and volume distribution. In addition to platelet count, platelet indices, including mean platelet volume and platelet distribution width, were automatically measured. The blood sample is diluted and passed through the apparatus, where changes in electrical resistance generated by individual cells produce pulses that are counted to determine the number and size of blood cells. The device uses hydrodynamic focusing and coincidence correction algorithms to reduce interference from platelet clumping, fragmented erythrocytes, and microcytic red blood cells in order to improve analytical accuracy. A leukocyte five‐part differential analysis was performed using flow cytometry with laser light–scattering technology, allowing differentiation of leukocyte subpopulations based on cell size, granularity, and internal complexity. All analyses were conducted in accordance with the standard operating procedures and manufacturer′s instructions [10, 11]. Standard laboratory operating procedures were followed for reviewing samples with flagged or abnormal blood cell values, and additionally, blood film morphology was assessed for cross‐checking the presence of discrepancies by Primo Star Microscope (Supporting Information 2: Annex S2 and Supporting Information 4: Annex S4).

#### 2.6.4. CD4

The BD FACS machine was used to determine CD4 count. The machine uses the principle of flow cytometry and an antibody tagged with fluorochrome by combining the hydrodynamic focusing, laser light scattering, and fluorescence detection to identify, quantify, and sort cells based on their physical and immunophenotypic characteristics to determine the CD4 rate. Fluorescently labeled antibodies attached to particular cell surface or intracellular antigens emit light at characteristic wavelengths that are detected by photomultiplier tubes, allowing precise multiparametric analysis and sorting of distinct cell populations such as CD4+ T lymphocytes. Cells suspended in a fluid stream are hydrodynamically focused to pass single‐file through one or more laser beams. As each cell intercepts the laser, it produces forward scatter (FSC), which is proportional to cell size, and side scatter (SSC), which is related to internal complexity or granularity.

#### 2.6.5. Viral Load Assay

HIV‐1 viral load was measured using the Xpert HIV‐1 viral load assay on the GeneXpert platform (Cepheid, Sunnyvale, CA, United States), which utilizes real‐time reverse transcription polymerase chain reaction (RT‐PCR) in a fully automated, cartridge‐based system. The plasma was loaded into a single‐use cartridge where viral RNA was automatically extracted, purified, and reverse transcribed. The target viral nucleic acid was amplified using sequence‐specific primers, and amplification was monitored in real time through fluorescently labeled molecular probes. The emitted fluorescence is proportional to the amount of amplified product, allowing quantitative determination of viral load reported as copies per milliliter.

### 2.7. Quality Management of Data and Laboratory Procedures

#### 2.7.1. Sociodemographic Data Quality Management

Prior to data collection, data collectors received training to ensure the reliability and validity of the data and to minimize technical and observer bias. The questionnaire was pretested on a random sample of patients from the study site to assess its reliability and validity. To ensure accuracy in translation, the questionnaire was reviewed by three individuals and back‐translated from Amharic to English. The validity of the final instrument was checked again (Supporting Information 1: Annex S1 and Supporting Information 2: Annex S2).

#### 2.7.2. Data Management and Analysis

Data were entered into EpiData Version 4.6 (EpiData Inc., Redwood City, CA, United States) and analyzed using STATA Version 14 (statistical software for statistics and data science) developed by Stata Corp for data (Stata Corp, College Station, TX, United States) after daily checks for completeness, accuracy, and consistency by the principal investigator. Hematological parameters were interpreted using standard clinical reference ranges, and variables including RBC count, Hgb, WBC count, platelet count, reticulocyte count, and immature reticulocyte fraction were categorized as low, normal, or high, while immunological parameters such as viral load and CD4 count were categorized into three categories by each reference standard value. Normality of continuous variables was evaluated using histograms and the Shapiro–Wilk test, with normally distributed data summarized as mean ± SD and outliers excluded. Descriptive statistics, including frequencies, percentages, charts, and tables, were used to summarize the data. Chi‐square statistical tests and the Spearman correlation tests were done at a *p* value < 0.05. The measure of associations between variables was initially examined using bivariable logistic regression, and variables with *p* ≤ 0.25 were included in a multivariable logistic regression model to adjust for confounders. Statistical significance was defined as *p* < 0.05. Based on the study results, conclusions and recommendations were drawn.

#### 2.7.3. Result Dissemination

The study findings were submitted to the Department of Medical Laboratory Sciences and CMHS at MTUTH, as well as to the study site. The abstract would be shared with relevant local bodies, including EMLA and libraries. Additionally, the results would be disseminated to the research community through conference presentations and publication in peer‐reviewed journals to reach an international audience.

## 3. Results

### 3.1. Sociodemographic and Clinical Factors

A total of 358 participants were enrolled, of whom 216 (62.1%) were female. The median age was 38 years (interquartile range: 33–45) (Table 1).

**Table 1:** Sociodemographic and clinical factors of participants for magnitude and predictors of leukopenia and thrombocytopenia among HIV patients at MTUTH during 2023.

Variables	Category	Frequency	Percent (%)
Age	15–25	56	15.63
	25–35	63	27.6
	35–45	74	20.67
	45–60	92	25.7
	> 65	73	20.4

Sex	Male	145	40.45
	Female	213	59.55

Residency	Urban	235	65.73
	Rural	123	34.37

Marital status	Single	27	7.58
	Married	251	69.94
	Divorced	62	17.13
	Widowed	18	5.34

Occupation	Farmer	63	17.69
	Housewife	152	42.41
	Merchant	55	15.50
	Government employee	66	18.53
	Others	22	5.90

Educational status	No formal education	82	22.92
	Primary school	119	33.34
	Secondary school	101	28.37
	University/college	56	15.49

BMI	Normal	247	69.10
	Underweight	59	16.30
	Overweight	52	14.6

Malaria infection	Yes	62	17.31
	No	296	82.69

Pregnancy	Yes	53	14.80
	No	305	85.20

Gestational age	1^st^	17	31.61
	2^nd^	14	26.65
	3^rd^	22	41.74

MTB infection	Yes	23	6.42
	No	335	93.58

Cigarette smoking	Yes	67	18.71
	No	291	81.29

Hypertension	Yes	109	30.44
	No	153	42.73
	I do not know	96	26.81

Alcohol consumption	Yes	165	46.13
	No	193	53.87

History of transfusion	Yes	31	8.66
	No	327	91.34

Stage of AIDS (disease) (*n* = 358)	Stage 1	278	77.65
	Stage 2	60	16.76
	Stages 3 and 4	20	5.59

History of neoplastic disease (*n* = 358)	Yes	24	6.70
	No	334	93.30

Viral load	≤ 1000	329	90.80
	> 1000	29	9.20

CD4	< 200	76	21.23
	≥ 200	282	78.77

Type of HAART	1C (AZT‐3TC‐NVP)	53	14.80
	1E (TDF‐3TC‐EFV)	167	46.65
	1J (TDF + 3TC + DTG)	77	21.51
	2H (TDF + 3TC + NVP)	24	6.70
	Others (1F + 2I + 1D)	37	10.34

HAART—Regimen	AZT‐containing	81	22.63
	Non‐AZT	277	77.37

*Note:* 1D, zidovudine–lamivudine–efavirenz; 1F, tenofovir disoproxil fumarate + lamivudine + dolutegravir; 2I, abacavir + lamivudine + lopinavir/ritonavir.

Abbreviations: 3TC, lamivudine; AZT, zidovudine; AZT, zidovudine containing regimen; CD4, cluster of differentiation 4; DTG, dolutegravir; EFV, efavirenz; HAART, highly active antiretroviral therapy; MTB, mycobacterium TB infection; NVP, nevirapine; TDF, tenofovir disoproxil fumarate.

### 3.2. Hematological and Immunological Profiles

The mean Hgb level of the study participants was 13.17 g/dL (95% CI: 12.99, 13.36) and 354 (332.40, 377), and the mean CD4+ was 354.84 (95% CI: 332.40, 377.29) and 0.34 (95% CI: 0.33, 0.35) (Table 2).

**Table 2:** Hematological and immunological profiles of study participants for magnitude and predictors of leukopenia and thrombocytopenia among HIV patients at MTUTH during 2023.

Parameters	With leukopenia (mean±SDat 95% CI)	Without leukopenia (mean±SDat 95% CI)	*p*value	With thrombocytopenia (mean±SDat 95% CI)	Without thrombocytopenia (mean±SDat 95% CI)	*p* value
WBC (×10^9^/L)	3.8 ± 1.74	7.8 ± 2.54	0.001	4.2 ± 2.35	5.34 ± 1.84	0.31
RBC (×10^12^/L)	3.44 ± 1.64	4.14 ± 1.7	0.27	2.74 ± 1.81	3.84 ± 1.90	0.15
Hgb (g/dL)	12.18 ± 1.39	14.18 ± 1.58	0.03	11.68 ± 1.65	13.98 ± 1.78	0.023
Platelet (×10^9^/L)	275 ± 125	255 ± 90	0.07	175 ± 105	275 ± 129	0.001
Neutrophil (×10^9^/L)	2.78 ± 1.75	3.34 ± 2.15	0.21	3.78 ± 1.85	2.74 ± 1.95	0.01
Eosinophilia (×10^9^/L)	0.31 ± 0.047	0.54 ± 0.14	0.06	0.51 ± 0.13	0.34 ± 0.15	0.34
Basophil (×10^9^/L)	0.04 ± 0.03	0.09 ± 0.05	0.11	0.05 ± 0.03	0.2 ± 0.07	0.59
Lymphocyte (×10^9^/L)	1.2 ± 0.86	2.1 ± 0.96	0.015	1.8 ± 1.10	1.3 ± 0.56	0.035
Monocyte (×10^9^/L)	0.57 ± 0.32	0.76 ± 0.46	0.04	0.87 ± 0.22	0.56 ± 0.36	0.62
CD4+ (cells/mm^3^)	318.14 ± 175.14	387.83 ± 205.02	0.03	251.05 ± 135.00	323 ± 185.02	0.003
Viral load (copies/mL)	919.77 ± 636	609.60 ± 506	0.005	821.70 ± 345	509 ± 307	0.006
Lymphocyte‐to‐WBC ratio	0.64 ± 0.24	0.27 ± 0.44	0.021	0.93 ± 0.11	0.47 ± 0.24	0.07
Lymphocyte‐to‐neutrophil ratio	0.9 ± 0.47	0.4 ± 0.27	0.03	1.04 ± 0.47	0.7 ± 0.28	0.002

Abbreviations: CD, cluster of distribution; RBC, red blood cell; WBC, white blood cell.

### 3.3. Correlation of Hematological Parameters and Viral Load With the CD4

The Hgb, lymphocyte, and lymphocyte‐to‐WBC ratio had a positive correlation with CD4+, but viral load had a negative correlation with CD4+. All parameters mentioned above had a weak and significant correlation (*p* < 0.05). However, the lymphocyte‐to‐neutrophil ratio to CD4+ had no significant correlation (Table 3).

**Table 3:** Correlation of hematological parameters and viral load with CD4 of study participants for magnitude and predictors of leukopenia and thrombocytopenia among HIV patients at MTUTH during 2023.

Parameters	Correlation coefficient	Strength (direction)	*p* value
Hgb to CD4+	0.4	Weak (positive direction)	0.045
Lymphocyte to CD4+	0.16	Weak (positive direction)	0.003
Viral load to CD4+	−0.20	Weak (negative direction)	0.0005
Lymphocyte‐to‐WBC ratio to CD4+	0.12	Weak (positive direction)	0.005
Lymphocyte‐to‐neutrophil ratio to CD4+	0.006	Weak (positive direction)	0.29

Abbreviations: CD, cluster of distribution; Hgb, hemoglobin; RBC, red blood cell; WBC, white blood cell.

### 3.4. Hematological Abnormalities Among HIV Patients

The magnitude of leukocytopenia, leukocytosis, thrombocytopenia, pancytopenia, and anemia, respectively, among the HIV patients was 37 (10.34%, 95% CI: 8.34, 14.34), 12 (3.35%, 95% CI: 2.15, 5.45), 53 (14.8%, 95% CI: 11.75, 17.85), 10 (2.8%, 95% CI: 1.08, 5.81), and (36.9%, 95% CI: 34.90, 40.90).

### 3.5. Predictors of Leukocytopenia and Thrombocytopenia

Bivariate and multivariable logistic regression models were used to assess the association of leukopenia and thrombocytopenia with independent variables. Variables with *p* < 0.25 in the bivariable analysis were included in the multivariable model, and associations with *p* < 0.05 in the multivariable analysis were considered statistically significant. Accordingly, age greater than 65, pregnancy, hypertension, history of alcohol consumption, and being underweight and overweight showed a significant association with leukopenia (Table 4), whereas age greater than 65, pregnancy, hypertension, and being underweight and being overweight showed a significant association with thrombocytopenia (Table 5).

**Table 4:** Bivariable and multivariable logistic regression model analyses of factors associated with leukocytopenia among patients with HIV at MTUTH (*N* = 358).

Variables	Category	Leukocytopenia		COR at CI (95%)	AOR at CI (95%)	*p* value
		Yes	No			
Age	15–25	4 (7.14%)	52 (92.86%)	1	1	
	25–35	7 (10.00%)	56 (90.00%)	1.59 (1.29, 1.79)	1.69 (0.67, 2.69)	0.04
	35–45	6 (8.1%)	68 (91.8%)	1.14 (0.94, 1.34)	0.86 (0.56, 1.16)	0.06
	45–65	8 (8.70%)	84 (91.30%)	1.24 (1.04, 1.44)	1.44 (1.34, 1.54)	0.12
	> 65	12 (16.44%)	61 (83.56%)	2.51 (2.15, 3.95)	2.80 (1.41, 3.36)	0.001

Sex	Male	14 (9.65%)	131 (90.65%)	1	1	
	Female	23 (10.85%)	190 (89.62%)	1.13 (0.56, 2.28)	1.07 (0.49, 2.30)	0.87

Residence	Urban	24 (10.21%)	211 (89.79%)	1	1	
	Rural	13 (10.60%)	110 (89.43%)	1.09 (0.51, 2.22)	1.04 (0.97, 1.11)	0.23

Pregnancy	Yes	14 (26.41%)	39 (73.59%)	4.40 (2.09, 9.27)	3.92 (1.76, 8.71)	0.001
	No	23 (7.54%)	282 (92.46%)	1	1	

Alcohol consumption	Yes	21 (12.72%)	144 (87.27%)	1.61 (0.81, 3.20)	2.1 (1.03, 2.83)	0.07
	No	16 (8.30%)	177 (91.70%)	1	1	

BMI	Normal	15 (5.81%)	243 (94.19%)	1	1	
	Underweight	13 (22.80%)	44 (77.2.0%)	4.77 (2.12, 10.70)	4.77 (2.12, 10.70)	0.0002
	Overweight	9 (20.93%)	34 (79.07%)	4.27 (1.73, 10.51)	3.62 (0.82, 3.92)	0.06

MTB infection	Yes	13 (23.64%)	42 (76.36%)	3.72 (1.76, 7.88)	3.67 (1.89, 7.12)	0.001
	No	24 (6.70%)	289 (93.30%)	1	1	

Viral load	≤ 1000	12 (41.4)	17 (58.60)	1	1	
	> 1000	25 (8.50)	304 (91.50)	8.59 (3.69, 19.96)	5.12 (3.01, 8.19)	0.046

CD4	< 200	21 (27.63)	55 (72.7)	6.4 (3.11, 12.9)	4.32 (2.02, 7.45)	0.041
	≥ 200	16 (5.67)	266 (94.33)	1		

Hypertension	Yes	20 (18.35)	89 (81.65)	3.06 (1.56, 6.21)	3.56 (1.96, 6.986)	0.0015
	No	17 (6.83)	232 (93.17)	1		

Abbreviations: BMI, body mass index; CD4, cluster of differentiation; MTB, mycobacterium tuberculosis.

**Table 5:** Bivariable and multivariable logistic regression model analyses of factors associated with thrombocytopenia among patients with HIV at MTUTH (*N* = 358).

Variables	Category	Thrombocytopenia		COR at CI (95%)	AOR at CI (95%)	*p*value
		Yes	No			
Age	15–25	7 (12.50%)	49 (87.50%)	1	1	
	25–35	9 (13.85%)	55 (86.15%)	1.12 (0.34, 3.24)	0.98 (0.78, 1.18)	0.06
	35–45	12 (17.90%)	62 (82.10%)	1.29 (0.47, 3.52)	0.75 (0.43, 1.12)	0.14
	45–65	11 (12.64%)	80 (83.36%)	1.01 (0.37, 2.79)	0.98 (0.58, 1.38)	0.07
	> 65	14 (19.18%)	59 (80.82%)	1.66 (0.62, 4.40)	2.20 (1.02, 3.72)	0.009

Pregnancy	Yes	16 (37.21%)	37 (62.79%)	5.84 (5.34, 6.34)	4.76 (2.11, 10.72)	0.001
	No	37 (6.89%)	268 (93.11%)	1	1	

History of hypertension	Yes	21 (18.92%)	90 (81.08%)	4.8 (3.18, 5.98)	3.92 (1.98, 7.77)	0.001
	No	7 (4.83%)	144 (91.66%)	1	1	
	I do not know	9 (5.52%)	87 (94.47%)	2.12 (1.07, 3.05)	1.43 (0.96, 1.83)	0.09

Alcohol consumption	Yes	27 (4.82%)	138 (95.18%)	3.34 (2.74, 3.64)	2.85 (1.5, 4.74)	0.001
	No	26 (5.52%)	167 (94.48%)	1	1	

BMI	Normal	23 (6.41%)	234 (93.59%)	1	1	
	Overweight	11 (13.95%)	31 (96.05%)	2.3 (2.10, 2.70)	1.740 (0.88, 2.88)	0.007
	Underweight	19 (23.73%)	40 (66.27%)	4.4 (4.01, 4.81)	3.61 (1.88, 6.93)	0.001

TB infection	Yes	11 (20.00%)	44 (80.00%)	3.2 (2.80, 3.40)	2.74 (1.39, 5.40)	0.004
	No	42 (7.26%)	261 (92.74%)	1	1	

Viral load	≤ 1000	14 (48.30)	15 (51.7)	1		
	> 1000	39 (11.85)	290 (88.15)	6.94 (3.11, 15.46)	4.98 (1.02, 3.84)	0.043

CD4	< 200	24 (31.58)	52 (68.42)	4.03 (2.17, 7.46)	3.61 (1.53, 6.56)	0.012
	≥ 200	29 (10.82)	253 (89.18)	1		

Abbreviations: BMI, body mass index; CD4, cluster of differentiation; MTB, mycobacterium tuberculosis.

## 4. Discussion

HIV is an RNA virus that progressively weakens the immune system, primarily by targeting and destroying certain blood cells, especially T‐helper lymphocytes (CD4+ cells). The hematological and immunological profiles decrease due to direct and indirect suppression of the bone marrow, leading to a reduction in multiple cell lines. CD4 cell count remains a key parameter in HIV/AIDS patients for monitoring immune status and disease progression. On the other hand, hematological indices, such as Hgb content, LNR, and LLR, are easily accessible markers of immunological state and systemic inflammation. When taken as a whole, these metrics offer important information about inflammatory load and immunological failure in HIV‐positive individuals [3, 12].

The magnitude of leukocytopenia, thrombocytopenia, and pancytopenia, respectively, among the HIV‐infected adults were 10.34% (95% CI: 8.34, 14.34), 14.8% (95% CI: 11.75, 17.85), and 2.8% (95% CI: 1.08%, 5.81%).

According to this study, the magnitude of leukocytopenia was 10.34% (95% CI: 8.34, 14.34). The results of this study were lower than those reported in other regions of Ethiopia (Jimma [13%] [6], Shewa [13.9%] [13], Dessie [24.4%] [14], and Gondar [35.6%] [15]), Nigeria (26.8%) [16], Uganda (24.3%) [17], India (18.33) [18], Tanzania (23.6) [19], Jounde (Cameroon) (34.68) [20], China (33.2%) [21], and the United States (Carolina) (43.4) [22]. The presence of coinfection and coexisting opportunistic infections that cause immunosuppression and require chemotherapy, such as some antibiotics (chloramphenicol), which lowers leukocyte counts, might have been the reason for the high level of leukopenia in the mentioned studies [23]. The findings of this study were higher than those reported in studies conducted in Gondar (Ethiopia) (6%) [24] and Nigeria (6.1%) [25]. The higher prevalence may reflect low access to HAART, late initiation of treatment, and lower virological suppression among participants, which are known to reduce HIV‐related bone marrow suppression. The reason for the decrement of leukopenia among HIV patients is due to better adherence of HIV patients than our study population, who had poor adherence to ART follow‐up, resulting in immunocompromising and low leukocyte counts [14]. The observed inconsistencies may have been caused by differences in study population characteristics, illness stage, HAART coverage, HAART duration, geographic, socioeconomic, and nutritional status, as well as sample size variations among studies.

The factors associated with leukopenia were age greater than 65 (2.80, 95% CI: 1.41, 3.36), pregnancy (3.92, 95% CI: 1.76, 8.71), being underweight (4.77, 95% CI: 2.12, 10.70), TB infection (3.67, 95% CI: 1.89, 7.121), history of alcohol consumption (2.10, 95% CI: 1.03, 3.83), viral load > 1000 copies/mL (5.12, 95% CI: 3.01, 8.19), and CD4 + <200/*μ*L of blood (4.32, 95% CI: 2.02, 7.43).

According to the findings of our study, HIV patients who were older than 65 were 2.80 times more vulnerable to leukopenia than the younger ones. This finding is supported by studies done in the United States [26] and Spain [26]. The reason for high leukocytopenia in geriatric HIV patients might be age‐associated immune suppression that results in bone marrow suppression and a decreased number of leukocytes due to the replacement of the hematopoietic microenvironment with yellow adipose tissue, which has low cellularity [5].

This study revealed that pregnant women were 3.92 times more likely to develop leukopenia than other women. The findings of this study are in agreement with the study done in Côte d′Ivoire (Abidjan) [27]. The main cause for the high leukopenia among pregnant women might be associated with the low production rate of leukocytes due to pregnancy and the HIV‐correlated effect on those pregnant women with HIV [28].

The findings of this study indicated that hypertensive HIV patients are 3.56 times more likely to develop leukopenia than individuals without hypertension. The findings of this study contradict the study done at Harari, which indicates that leukocyte count has a direct correlation with hypertension. The possible reason for this contradiction is variation in the study population; the population of this study was HIV‐infected, but the study at Harar included a nonhypertensive population. In HIV‐infected hypertensive patients, leukopenia is directly correlated with HIV infection [29].

This study′s findings revealed that being underweight results in 4.77 times more vulnerability to leukopenia than that of normal DM patients. This study is supported by a study done in Paris [30]. The possible reason for the increased leukopenia among undernourished HIV patients might be due to ineffective hematopoiesis that resulted from a lack of essential micronutrients; consequently, infection can lead to malnutrition, in which poor nutritional status further impairs leucopoiesis [30, 31].

According to this study′s findings, HIV patients who were infected with MTB were 3.67 times more susceptible to leukopenia than those without MTB. These might be due to TB infection‐induced bone marrow suppression, which results in a decrease in myeloid progenitor cells, heightened immunological activation, and the combined impact of both diseases′ chronic inflammation. Furthermore, leukocyte production may be further suppressed by antiretroviral and antituberculosis medications, raising the likelihood of leukopenia in TB‐HIV coinfected individuals [24].

The study′s findings also indicated that people who use alcohol more than the standard were 2.1 times more vulnerable to leukopenia than others. The possible reason for this high leukopenia in alcoholic PLWHIV might be due to cessation of myelopoiesis, with respective suppression of VB12 malabsorption and alcohol‐associated liver disease, resulting in leukopenia [32].

The study found that HIV patients with a viral load above 1000 copies/mL were 5.12 times more likely to develop leukopenia compared to those with a viral load below 1000 copies/mL. The findings of this study were supported by studies done at Desie (Ethiopia) [14], Hawassa (Ethiopia) [33], and China [34]. The possible reason might be elevated viral load due to uncontrolled replication, which promotes immune activation, bone marrow suppression, and leukocyte destruction, resulting in leukopenia [32].

The HIV patients with a low CD4+ cell count (< 200/*μ*L of blood) were 4.32 times more likely to develop leukopenia than those with CD4 + >200/*μ*L of blood. This study′s findings were in agreement with studies done at Mehal Meda (Ethiopia) [35] and Uganda [17]. This might be due to advanced immunosuppression, as CD4+ levels decline, the risk of opportunistic infections and hematopoietic dysfunction increases, contributing to leukocyte depletion and poorer clinical outcomes [15].

This study revealed that individuals with leukopenia showed markedly decreased RBC, Hgb and lymphocyte count, CD4, and a higher viral load. This might be due to an increased suppression of hematopoiesis and immune cell production, resulting from increased viral number, indicating more advanced immune compromise and poorer virological control. The lymphocyte‐to‐WBC ratio and lymphocyte‐to‐neutrophil ratio were notably higher in participants with leukopenia, suggesting an alteration in leukocyte distribution that may reflect chronic immune activation and dysregulation [13, 36, 37].

The magnitude of thrombocytopenia among the HIV patients was 14.8% (95% CI: 13.75, 15.85). The finding of this study was comparable with a study done in the United States (Carolina) (15.5%) [22]. The finding of this study was greater than studies in different parts of Ethiopia (Jimma [7.8%] [6], Shewa [12.8%] [13], and Gondar [4.1%] [15]), Uganda (8.3%) [17], and China (13.6, 21) and lower than studies in Ethiopia (Dessie) (18.7%), Ethiopia (Gondar) (20%) [38], Lagos (Nigeria) (16.1%) [16], and Cameroon (27.1%) [20]. The possible reason for this variation might be the variation in adherence of HIV patients on HAART and the variation of antiretroviral therapy, as individuals who were on advanced HAART (dolutegravir‐based therapy) had good improvement, as well as a limited effect on myeloid progenitor cell production [39].

The factors associated with thrombocytopenia were age greater than 65 (2.2, 95% CI: 1.72, 2.78), pregnancy (4.1, 95% CI: 3.63, 4.67), hypertension (4.1, 95% CI: 3.63, 4.83), being underweight (5.42, 95% CI: 4.82, 5.92), alcohol consumption (4.21, 95% CI: 4.01, 4.31), viral load (6.90, 95% CI: 3.11, 15.46), and CD4+ (4.03, 95% CI: 2.17, 7.46).

As indicated in this study, PLWHIV who are older than 65 had a 2.2‐fold higher likelihood of thrombocytopenia compared to those under 65 years. The reason for high thrombocytopenia in geriatric patients might be due to the long‐term HIV‐related bone marrow suppression, age‐related immunological dysregulation, and hematopoietic loss. Additionally, the presence of acquired TTP, which increases platelet destruction, and chronic HIV‐associated bone marrow suppression among aged patients. The greater incidence of thrombocytopenia in this age group is probably due to the cohabitation of long‐term HIV infection and aging‐related marrow depletion [40].

This study revealed that pregnant women with HIV were four times more susceptible to thrombocytopenia than nonpregnant women. The possible reason might be gestational thrombocytopenia with co‐occurrence with HIV‐associated thrombocytopenia or the combined effect of gestational thrombocytopenia and HIV‐associated thrombocytopenia. HIV contributes to impairing megakaryocyte function, immune‐mediated platelet destruction, and chronic inflammation, whereas pregnancy produces a normal decrease in platelet count through hemodilution and increased platelet consumption. When these mechanisms overlap, platelet decrease is accelerated, making pregnant HIV‐positive women more vulnerable to thrombocytopenia [41].

This study also revealed that individuals who had hypertension were 4.1 times more likely to develop anemia than those individuals who were not hypertensive. The possible reason for this might be a high level of platelet consumption due to blood vessel narrowing and TTP. Additionally, hypertension can cause endothelial dysfunction and increased platelet activation as well as disseminated intravascular coagulation, leading to higher platelet consumption and lower circulating platelet counts [22].

The findings of this indicated that being underweight was 5.42 times more likely to develop thrombocytopenia than being of normal weight. This might be due to the reason that underweight was associated with worse inpatient outcomes among HIV‐infected patients. The underweight may be due to malnutrition (undernutrition), which impairs hematopoiesis and limits platelet production due to deficiencies in iron, folate, and vitamin B12, increasing the risk of thrombocytopenia.

This study′s findings indicated that people who consume more alcohol were 4.21 times more vulnerable to thrombocytopenia than others. The possible reason might be that alcohol may result in liver dysfunction, which lowers thrombopoietin synthesis and increases platelet destruction, as well as direct bone marrow suppression, which inhibits platelet production. Alcohol consumption increases the risk of thrombocytopenia in HIV‐positive individuals by exacerbating immunological suppression, interfering with adherence to antiretroviral medication, and amplifying HIV‐related hematological abnormalities [42].

The findings of this study showed that HIV patients with viral load > 1000 copies/mL are more likely to develop thrombocytopenia than those with lower viral load. The possible reason might be that higher viral replication leads to chronic immune activation, which triggers the production of inflammatory cytokines that can enhance platelet destruction, and HIV can also directly infect megakaryocytes, which reduces the bone marrow capacity to make platelets. The significant drop in platelet counts seen in patients with uncontrolled viremia is caused by both increased destruction and decreased generation [9, 31].

The study found that HIV patients with CD4+ counts below 200/*μ*L were 4.03 times more likely to develop thrombocytopenia compared to those with higher counts. This might be due to increased susceptibility to opportunistic infections in individuals with low CD4+ levels, some of which inhibit platelet formation either directly or indirectly due to the presence of advanced HIV illness–associated immunosuppression and persistent systemic inflammation that may harm bone marrow′s hematopoietic cells [43].

The thrombocytopenia exhibited significant reductions in RBC count and Hgb concentration, reflecting the coexistence of cytopenia. Thrombocytopenia was also associated with lower CD4+ cell counts and higher viral load levels compared with individuals without thrombocytopenia, further supporting its association with disease severity and immune dysfunction. Individuals with thrombocytopenia had relatively higher neutrophil and monocyte counts but reduced lymphocyte counts. The lymphocyte‐to‐WBC and lymphocyte‐to‐neutrophil ratios were notably higher in participants with thrombocytopenia, suggesting alterations in leukocyte distribution that may reflect chronic immune activation and dysregulation [44, 45].

Among the study population, 21.23%, 21.2%, 38.83%, 21.23%, and 18.72% had CD+ value < 200, 200–350, 350–500, and >500, respectively. The study′s findings indicated that 21.23% (95 CI: 18.95, 27.25) HIV patients were under severe immunosuppression, which is comparable with a study done in China (26.1%) [34], lower than the study done in different parts of the world, such as Cameroon [37]. The Hgb, lymphocyte, lymphocyte‐to‐neutrophil ratio, and lymphocyte‐to‐leukocyte ratio have a positive correlation with CD+ count, whereas viral load is negatively correlated with CD+. The findings of this study were in line with the studies done in Nigeria and Indonesia [4, 46], which state that NLR was positively correlated with CD+.

The findings of this study were also comparable with the findings of the study done in Bali (Indonesia) [47], in which the variables, which were positively correlated in this study, had a positive correlation with CD4+. The findings of this study were also supported by a study conducted in Brazil [48]. But it disagrees with the study done in India [3], which indicated that there is no correlation between the viral load and CD4+. The difference might be due to the age variation and variation in the study population.

## 5. Conclusion

Leukocytopenia and thrombocytopenia are public health problems, particularly among HIV patients. Leukopenia was significantly associated with older age (> 65 years), pregnancy, underweight status, tuberculosis infection, history of alcohol consumption, high viral load (> 1000 copies/mL), and severe immunosuppression (CD4+ <200/μL of blood), where as thrombocytopenia was significantly associated with pregnancy, hypertension, undernutrition, alcohol consumption, high viral load, and low CD4+ count. Hgb, lymphocyte, and lymphocyte‐to‐WBC ratio all have a weak positive correlation with CD4+; however, viral load has a negative correlation with CD4+.

## 6. Strength of the Study

This study provides important information about the prevalence and determinants of leukopenia and thrombocytopenia. Hematological indicators for HIV patients were also reported. This study gives useful information for resource‐limited environments in predicting HIV patients′ CD4 levels using the lymphocyte‐to‐neutrophil ratio, lymphocyte‐to‐leukocyte ratio, and Hgb values.

## 7. The Study Limitation

This cross‐sectional study may not provide complete information on cause and effect or temporal relationships. It also does not allow for causality in the link between leukopenia and thrombocytopenia and their determinants. This study did not focus on chronically hospitalized HIV patients′ hematological parameters and their correlation with CD4.

## 8. Recommendation

For PLWHIV/AIDS, routine hematological testing, particularly for leukocyte and thrombocyte counts, is advised, especially for older adults, pregnant women, and those with chronic illnesses. Early screening and management of hematological abnormalities, particularly leukocytopenia and thrombocytopenia, is recommended for PLWHIV, especially individuals with high viral load, low CD4+ count, tuberculosis, hypertension, undernutrition, and alcohol users, to reduce their burden. Nutritional support, optimized antiretroviral therapy, and integrated care for comorbid conditions should be prioritized to improve hematological outcomes. As thrombocytopenia and leukopenia are public health issues, medical professionals are encouraged to pay attention to them. In resource‐limited settings, Hgb, lymphocyte, lymphocyte‐to‐neutrophil ratio, and lymphocyte‐to‐leukocyte ratio should be used for the prediction of HIV severity.

**Nomenclature**
AIDS: acquired immunodeficiency syndrome
ART: antiretroviral therapy
LNR: lymphocyte‐to‐neutrophil ratio
LLR: lymphocytes‐to‐leukocyte ratio
CD: cluster of differentiation
CI: confidence interval
EDTA: ethylenediaminetetraacetic acid
HAART: highly active antiretroviral therapy
HIV: human immunodeficiency virus
PLWHIV: peoples living with human immunodeficiency virus
RBC: red blood cell
MTUTH: Mizan Tepi University Teaching Hospital
WHO: World Health Organization
WBC: white blood cell

## Author Contributions

S.S.K. conceived the idea. S.S.K., D.Z., T.B., and E.A. wrote the proposal. S.S.K., G.M.B., D.Z., E.A., and T.B. participated in the data collection and data analysis and assisted with manuscript drafting. E.A. and T.B. revised and commented on the design, analysis, and manuscript. G.M.B., T.B., D.Z., E.A., and S.S.K. designed, supervised data quality, participated in analysis, and prepared the manuscript. S.S.K., T.B., G.M.B., and T.B. reviewed, edited, and approved the manuscript. S.S.K. analyzed the data and led manuscript drafting. S.S.K. is the corresponding and first author of this article.

## Funding

No funding was received for this manuscript.

## Disclosure

The final draft of the manuscript for the publication was approved by all authors.

## Ethics Statement

The Mizan Tepi University College of Medicine and Health Sciences Research and Ethics Review Committee granted ethical permission for the study (Reference Number CMHS/2750/23), and all protocols followed the Declaration of Helsinki. Additionally, MTUTH granted permission to carry out the study. Children′s agreement was asked before participation, and parents or legal guardians provided written informed consent after being educated about the study′s goals, advantages, and possible hazards. As participation in the study was voluntary, participants were free to decline at any time.

## Consent

The authors have nothing to report.

## Conflicts of Interest

The authors declare no conflicts of interest.

## Supporting Information

Additional supporting information can be found online in the Supporting Information section.

## Supporting information


**Supporting Information 1** Annex I: English version information sheet and consent form. Part I: information sheet: includes study introduction, title, objectives, benefits, risks, privacy rights, and contact information for participants. Informed consent form: signed consent template documenting participant agreement for study participation. Sociodemographic, clinical, and nutritional questionnaire: data collection tool for participant characteristics, medical history, lifestyle, and nutritional habits.

**Supporting Information 2** Annex II: Dummy tables. Table S1: Hematological profiles (CBC parameters) of participants. Table S2: Age and sex distribution of participants. Table S3: Marital status and hematological abnormality distribution. Table S4: Occupation and hematological abnormality distribution. Table S5: Locality (urban/rural) and hematological abnormality distribution. Table S6: History of comorbidities, medication, physical activity, and hematological outcomes.

**Supporting Information 3** Annex III: Materials and reagents. Lists all laboratory consumables and reagents used in sample collection, CBC analysis, staining, and microscopy (e.g., hematology analyzer, Wright stain, needles, tubes, and reagents).

**Supporting Information 4** Annex IV: Principles and laboratory procedures. Venous blood collection protocol: stepwise procedure for safe and standardized blood sampling. Complete blood count (CBC) by hematology analyzer: principle, specimen requirements, and stepwise procedure for quantitative analysis of blood cells. Wright stain procedure: principle, staining method, and interpretation of peripheral blood film findings, including grading of fragmented cells, spherocytes, and nucleated RBCs.

## Data Availability

The first and corresponding author will provide the required data upon reasonable request.
