# Supplementary material for: Magnitude and Predictors of Leukopenia and Thrombocytopenia in Adults With HIV/AIDS Attending Mizan Tepi University Teaching Hospital, Southwest Ethiopia
Source: Biomed Res Int. 2026 Apr 10;2026:5907903. doi: 10.1155/bmri/5907903 (PMC13067301; doi:10.1155/bmri/5907903)
Supplement: Supplementary file 1 — Supporting Information 1 Annex I: English version information sheet and consent form. Part I: information sheet: includes study introduction, title, objectives, benefits, risks, privacy rights, and contact information for participants. Informed consent form: signed consent template documenting participant agreement for study participation. Sociodemographic, clinical, and nutritional questionnaire: data collection tool for participant characteristics, medical history, lifestyle, and nutritional habits. [file BMRI-2026-5907903-s004.pdf]

Annex I: English Version Information sheet and Consent form

Part I: Information sheet

Introduction: Here we are academic staff and going to conduct a survey Magnitude and Predictors of Leukopenia and Thrombocytopenia in Adults with HIV/AIDS Attending Mizan Tepi University Teaching Hospital, Southwest Ethiopia.

Study title: Magnitude and Predictors of Leukopenia and Thrombocytopenia in Adults with HIV/AIDS Attending Mizan Tepi University Teaching Hospital, Southwest Ethiopia.

Objective of the study: The aim of this study is to determine Magnitude and Predictors of Leukopenia and Thrombocytopenia in Adults with HIV/AIDS Attending Mizan Tepi University Teaching Hospital, Southwest Ethiopia, 2023 GC.

Benefit of this study: Conducting this study will be used to advance the diagnosis of anemia, leukopenia, leukocytosis and thrombocytopenia in these individuals and know the current status of the problems. If you have abnormal parameter, you will be linked to clinicians with your laboratory results for additional diagnosis and treatment of disease. The study output (result) will be used for planning health programs and policy makers for reduction of hematological abnormality. You understand that you will not get any financial benefit. Your cooperation and willingness to the study will be very helpful in understanding current prevalence of problem.

Risks of this study for participants: There may be little pain during blood sample collection but do not cause any health harm or long live abnormality.

Rights and privacy: Your name will not be written in the form and I assure you that all the information will be kept strictly confidential. Your participation is voluntary based and you are not obligated to participate. You should know that the information and blood sample that going to be used for this study only. All the information given for the study and the results are confidential. If you are not comfortable, please feel free to refuse. Therefore, with full understanding of the situations you agree to give the entire necessary information and blood sample for laboratory analysis.

Person to contact; please direct any questions or problems you may encounter during this study to the principal investigator. Address +251917768545/samissahile45@gmail.com

Informed consent

I participant undersigned the purpose of the study titled as Magnitude and Predictors of Leukopenia and Thrombocytopenia in Adults with HIV/AIDS Attending Mizan Tepi University Teaching Hospital, Southwest Ethiopia 2023. have been informed there is no harm except little discomfort during sample collections. I have been informed that other people will not know my results. I understand that there is no benefit to me personally apart from clinical service I get from these results. I have been told that participation in this study is voluntary and I may refuse to be in the study. The study has been explained to me in the language I understand. I give consent to participate after a clear understanding of the objectives and conditions of the study.

Participant's name ---------------------------Signature---------------------- Date: ----------------------

Data collector name: -------------------------- Signature: --------------------- Date: ----------------

Name of investigator _____________Signature ___________Date of investigation _______

ID.No:______________

Socio –demographic, clinical and nutritional information of diabetic adult patients

S no	Socio –demographic questions please encircle in correct information you have.		
01	How old are you? Age in years		
02	Sex	Male	1
		Female	2
03	Where do you live? (Residence)	Urban	1
		Rural	2
04	What is your occupation?	Farmer	1
		House wife	2
		Merchant	3
		Governmental employee	4
		Labor worker	5
		Others	6
		Orthodox	1
		Muslim	2
		Protestant	3
		Others	4
06	How much is your monthly income?		
07	What is your educational Status?	No formal education	1

		Primary School	2
		Secondary school	3
		University/college	4
08	Marital status	Single	1
		Married	2
		Divorced	3
		Widowed	4

B) Clinical data of participant

09	If Q2 is female	Pregnant	1
		Not pregnant	2
10	Recent blood loss	Yes	1
		No	2
11	History of transfusion	Yes	1
		No	2
12	If yes in No 9, how long it was? (in month)		
13	Another infection parasitic or bacterial	Yes<br>No	1<br>2
14	Hypertension	Yes	1
		No	2
16	Did you do daily physical activity	Yes	1
		No	2
		I don't know	3
19	Are you taking drug for DM?	Yes	1
		No	2
20	If Question number 19 yes then, how long		
21	If Question number Q 19 yes, did you take additional drug?		
22	Height		
23	Weight		
	BMI		
Nutrional data of participant			
24	Did you drink tea or coffee after meal?	Yes	1
		No	2
25	Do you eat meat	Yes	1
		No	2
26	If answer for question 25 is yes how many times	Daily	1
		Every two day	2
		Every two week	3
		Once a month	4

27	Do you eat vegetable	Yes	1
		No	2
28	If answer for question 27 is yes how many times	Daily	1
		Every two day	2
		Every two week	3
		Once a month	4
29	Have you taken iron /folate	yes	1
		No	2
