# Supplementary material for: Magnitude and Predictors of Leukopenia and Thrombocytopenia in Adults With HIV/AIDS Attending Mizan Tepi University Teaching Hospital, Southwest Ethiopia
Source: Biomed Res Int. 2026 Apr 10;2026:5907903. doi: 10.1155/bmri/5907903 (PMC13067301; doi:10.1155/bmri/5907903)
Supplement: Supplementary file 2 — Supporting Information 2 Annex II: Dummy tables. Table S1: Hematological profiles (CBC parameters) of participants. Table S2: Age and sex distribution of participants. Table S3: Marital status and hematological abnormality distribution. Table S4: Occupation and hematological abnormality distribution. Table S5: Locality (urban/rural) and hematological abnormality distribution. Table S6: History of comorbidities, medication, physical activity, and hematological outcomes. [file BMRI-2026-5907903-s003.pdf]

Annex II; Dummy table

Table I: - Age and Sex distribution of Magnitude and Predictors of Leukopenia and Thrombocytopenia in Adults with HIV/AIDS Attending Mizan Tepi University Teaching Hospital, Southwest Ethiopia, 2023

Age	Sex		Total
	Male	Female	
15-30			
31-45			
41-64			
>64			
Total			

Table S II; - Distribution of Magnitude and Predictors of Leukopenia and Thrombocytopenia in Adults with HIV/AIDS Attending Mizan Tepi University Teaching Hospital, Southwest Ethiopia, 2023

Sr. No	Marital status	Hematological abnormality		Total
		Positive	Negative	
1	Married			
2	Single			
3	Divorced			
4	Widowed			
Total				

Table S III: Distribution of Magnitude and Predictors of Leukopenia and Thrombocytopenia in Adults with HIV/AIDS Attending Mizan Tepi University Teaching Hospital, Southwest Ethiopia, 2022

Sr. No	Occupation	Hematological abnormality		Total
		Positive	Negative	
1	Student			
2	Employer			
3	House wife			
4	Laborer			
5	Farmer			
6	Others			

Table S IV; - Distribution of Magnitude and Predictors of Leukopenia and Thrombocytopenia in Adults with HIV/AIDS Attending Mizan Tepi University Teaching Hospital, south west Ethiopia, 2023

Sr. No	Locality	Hematological abnormality		Total
		Positive	negative	
1	Urban			
2	Rural			
3	Total			

Table SVII; - Distribution of Magnitude and Predictors of Leukopenia and Thrombocytopenia in Adults with HIV/AIDS Attending Mizan Tepi University Teaching Hospital, Southwest Ethiopia, 2023

History	**Yes**	**No**	**Total**
Past opportunistic illnesses			
Hypertension			
Type of Medication (antibiotics)			
Recent blood loss			
Duration of DM			
Physical activity			
