# Supplementary material for: Magnitude and Predictors of Leukopenia and Thrombocytopenia in Adults With HIV/AIDS Attending Mizan Tepi University Teaching Hospital, Southwest Ethiopia
Source: Biomed Res Int. 2026 Apr 10;2026:5907903. doi: 10.1155/bmri/5907903 (PMC13067301; doi:10.1155/bmri/5907903)
Supplement: Supplementary file 3 — Supporting Information 3 Annex III: Materials and reagents. Lists all laboratory consumables and reagents used in sample collection, CBC analysis, staining, and microscopy (e.g., hematology analyzer, Wright stain, needles, tubes, and reagents). [file BMRI-2026-5907903-s002.pdf]

Annex III; Materials and reagents

- Cotton
- Pencil
- Absolute Ethanol
- UnicelDxH800 hematology analyzer
- Glove
- Test tube
- 3- or 4-ml purple vacationer tube
- Needle
- Vacationer holder
- Alcohol swab (70%)
- Cotton balls
- Normal saline
- UnicelDxH800 reagent.
- Mixer
- New methylene /Brilliant Cresol Blue
- Antihumangloblin reagent.
- Wright satin
- Heparinized/EDTA capillary tube
- Disposable glove
- Tourniquet
- Microscope with 100X objective
- Microscope slides
- Glass test tubes
- Centrifuge
- Plastic pasture pipette
- Distilled water
- Water bath
- Oil immersion
