# Supplementary material for: Magnitude and Predictors of Leukopenia and Thrombocytopenia in Adults With HIV/AIDS Attending Mizan Tepi University Teaching Hospital, Southwest Ethiopia
Source: Biomed Res Int. 2026 Apr 10;2026:5907903. doi: 10.1155/bmri/5907903 (PMC13067301; doi:10.1155/bmri/5907903)
Supplement: Supplementary file 4 — Supporting Information 4 Annex IV: Principles and laboratory procedures. Venous blood collection protocol: stepwise procedure for safe and standardized blood sampling. Complete blood count (CBC) by hematology analyzer: principle, specimen requirements, and stepwise procedure for quantitative analysis of blood cells. Wright stain procedure: principle, staining method, and interpretation of peripheral blood film findings, including grading of fragmented cells, spherocytes, and nucleated RBCs. [file BMRI-2026-5907903-s001.pdf]

Annex IV; - Principles and Laboratory procedure

1. Sample procedure

Venous blood collection

1. Assemble all necessary equipment
2. Identify the patient and label the test tubes by patient's identification number.
3. Visually inspect and choose the arm from cephalic, basilic, and median cubital veins that was not repeatedly used for venipuncture, free of bruises, abrasions, and sites of infection.
4. Apply the tourniquet
5. Using a cotton ball saturated with 70% alcohol clean the skin in the area of the venipuncture.
6. Allow the site to dry
7. Use one hand to hold the evacuated tube or syringe and one or more fingers of the other hand to secure the skin area of the forearm below the intended venipuncture site.
8. Hold the needle with attached syringe or evacuated tube at angle of 20°about 1 to 2 inches below and in a straight line with the intended venipuncture site.
9. Gently insert the needle through the skin and into the vein.
10. Release tourniquet as soon as the blood begins to flow into the evacuated tube
11. After the desired amount of blood has been drawn place a gauze pad over the venipuncture site.
12. Withdraw the blood collecting unit with one hand and immediately press down on the gauze pad and elevate the entire arm.
13. Place a non-allergenic adhesive spot or strip over the venipuncture site
14. Mix tubes with anticoagulant by inverting the tubes several times.
15. Clean up supplies from the work area, remove gloves, and wash hands

2. Complete Blood Count by hematology analyzer (symiens)

Principle

The symiens ADVIA hematological analyzer is a quantitative, automated hematology analyzer for in-vitro diagnostic use in screening patient populations in clinical laboratories. The blood sample, which is suspended in diluted sample, will pass through the apparatus causing DC resistance. As this occur change in blood cell size is detected as the electrical pulse and blood cell count is calculated by counting pulse. The symiens ADVIA analyzer provides the following: CBC, Leukocyte 5-Part Differential (Diff), Reticulocyte, and Nucleated RBC on whole blood. A cyanide-free CBC lytic reagent that lyses RBC for the white blood cell count, and works in conjunction with Coulter DxH diluent to generate a stable hemoglobin measurement and used to lyse the RBC and discriminates nucleated RBC from white blood cells.

The reticulocyte stain reagent is a cyanide-free reagent that uses a dye to stain reticulocytes. The reticulocyte-clearing reagent is a cyanide-free reagent that stabilizes the dye-reticulum complex to enhance discrimination of reticulocytes from mature RBC utilizing the volume, conductivity and scatter technology. DxH Cleaner is a cyanide-free, aldehyde-free cleaning agent that degrades residual materials so that they may be flushed from the system with the diluent.

Specimen requirements

About 3-4 ml of venous blood collected into EDTA tubes.

Procedure

1. Turn on the power switch on the front side of the analyzer.
2. Perform quality control analysis on 3 levels of control blood material (low, normal and high) to verify that the instrument is performing within the specified ranges
3. Specimen will be collected into EDTA (purple) vacutainer (2 or 3 ml volume).
4. Well mix blood with EDTA and perform CBC and reticulocyte count.
5. After the specimen processing module cycles the samples, review the sample results at the system manager.
6. Finally print the output and register

3. Wright stain

Principle for Wright stain: -

Wright's stain is a polychromatic stain consisting of a mixture of eosin and methylene Blue. When applied to blood cells, the dyes produce multiple colors based on the ionic charge of the stain and the various components of the cell. The eosin ions are negatively charged and stain basic cell components an orange to pink color. The methylene blue ions are positively charged and stain the acid cell components in varying shades of blue. The neutral components of the cell are stained by both components of the dye producing variable colors.

Procedure

1. Place the air-dried blood smears with the smeared side upward on a horizontal staining rack.

2. Cover the blood smears completely with Wright's stain solution and let it remain for 1 -3 minute.

3. Wash the stain off with running tap water until the smear appears pink.

4. Remove the stain on the back of the smears by cleaning with alcohol-moistened gauze and dry.

5. Examine under a microscope.

Blood film finding interpretation: - The presence of <1% fragments in the PBF is considered normal and 1-5(+1), 6–14(+2) and >15 % (+3) finding of fragmented cell in blood film is reported in graded manner in bracket. However, the finding of even a single spherocyte and nucleated RBC is considered as indicators since, they are usually absent in the blood films of healthy individuals. Reporting and interpretation for spherocyte can be as follows, 1-5, 6–19, >20% finding of in blood film reported in grade as +1, +2 and +3 respectively.

Laboratory results ID.No:______________

Hematologic profiles	Mean	SD	Maximum	Minimum
Total leukocyte				
Neutrophil				
Eosinophil				
Basophil				
Monocyte				
Lymphocyte				
Erythrocyte				
HCT (L/L)				
MCV,fl				
MCH, pg				
MCHC, g/dl<br>RDW, f l<br>Platelet				
